# Malaria outbreak investigation in a rural area south of Zimbabwe: a case–control study

**DOI:** 10.1186/s12936-020-03270-0

**Published:** 2020-06-01

**Authors:** Paddington T. Mundagowa, Pugie T. Chimberengwa

**Affiliations:** 1 The Clinical Research Center, Africa University, 132 H. Chitepo Street, Mutare, Zimbabwe; 2 Ministry of Health and Child Care, P.O Box 441, Bulawayo, Matabeleland North Province Zimbabwe

**Keywords:** Malaria outbreak, Eaves, Case–control, Housing construction, Beitbridge

## Abstract

**Background:**

Ninety percent of the global annual malaria mortality cases emanate from the African region. About 80–90% of malaria transmissions in sub-Saharan Africa occur indoors during the night. In Zimbabwe, 79% of the population are at risk of contracting the disease. Although the country has made significant progress towards malaria elimination, isolated seasonal outbreaks persistently resurface. In 2017, Beitbridge District was experiencing a second malaria outbreak within 12 months prompting the need for investigating the outbreak.

**Methods:**

An unmatched 1:1 case–control study was conducted to establish the risk factors associated with contracting malaria in Ward 6 of Beitbridge District from week 36 to week 44 of 2017. The sample size constituted of 75 randomly selected cases and 75 purposively selected controls. Data were collected using an interviewer-administered questionnaire and Epi Info version 7.2.1.0 was used to conduct descriptive, bivariate and multivariate analyses of the factors associated with contracting malaria.

**Results:**

Fifty-two percent of the cases were females and the mean age of cases was 29 ± 13 years. Cases were diagnosed using rapid diagnostic tests. Sleeping in a house with open eaves (OR: 2.97; 95% CI 1.44–6.16; p < 0.01), spending the evenings outdoors (OR: 2.24; 95% CI 1.04–4.85; p = 0.037) and sleeping in a poorly constructed house (OR: 4.33; 95% CI 1.97–9.51; p < 0.01) were significantly associated with contracting malaria while closing eaves was protective (OR: 0.45; 95% CI 0.20–1.02; p = 0.055). After using backward stepwise logistic regression, sleeping in a poorly constructed house was associated with five-fold odds of getting sick from malaria (AOR: 8.40; 95% CI 1.69–41.66; p = 0.009). Those who had mosquito nets did not use them consistently. The district health team and the rural health centre were well prepared to response despite having limited human resources.

**Conclusion:**

Health promotion messages should emphasize the importance of closing the entry points of the malaria vector, and the construction of better houses in the future. Residents had to be educated in the importance of consistent use of mosquito nets. The district had to improve malaria preventive measures like distribution of mosquito nets and lobby for more human resources to assist with malaria surveillance thus, curbing the recurrence of malaria outbreaks.

## Background

Nearly half the global population is exposed to malaria [1] and in 2018, an estimated 228 million malaria cases occurred globally (95% Confidence interval (CI) 206–258 million) [2], up from 212 million in 2015 [3]. 405,000 malaria deaths were recorded in 2018 with the sub-Saharan region and India carrying 85% of the global malaria burden [2]. Malaria disease burden in Africa is very high and 90% of the global annual malaria mortality cases come from the African region [3]. In sub-Saharan Africa, 80–90% of malaria transmissions primarily occurs indoors during the night [4].

In Zimbabwe, malaria is a major public health problem affecting all age-groups and according to the World Health Organization (WHO) malaria report of 2016, 79% of the population are at risk of contracting the disease. Malaria incidence in the country was 139 per 1000 population at risk in 2013 [5]. The Ministry of Health and Child Care reported a surge in the malaria case fatality rate from 6.1% in 2012 to 13.8% in 2014 [6] and the malaria mortality cases were being reported throughout the year although transmission of the disease usually happens in seasonal epidemics largely occurring during the summer months of November to April [7].

The Zimbabwe National Malaria Control Programme and Roll Back Malaria Programme have achieved major successes in combating the diseases over the last 13 years, and this led to the reorientation of focus from malaria control to elimination [8]. These two programmes whose leadership is centrally stationed in the capital city, Harare, are informed by provincial and disease control officers who gets information from the district health teams which oversees malaria case management, surveillance and outbreak response at a local level [9]. The progress in malaria elimination was tenaciously challenged by vector and parasite resistance to insecticides and anti-malarial medicines, outdoor malaria transmission, dynamic vector behaviour and invasion of new areas by vectors. Climate change, erratic funding, local economic and political disturbances as well as increased cross border population movements have also contributed to the regression of malaria control programmes [8]. These factors have contributed to sporadic malaria outbreaks throughout Zimbabwe.

In 2017, the Beitbridge District was experiencing a second malaria outbreak in a space of less than 12 months. The first malaria outbreak in the district started in late 2016 and was declared over in week 22 of 2017. This earlier malaria outbreak recorded over 800 cases resulted in five fatalities, three children and two adults [10]. The recent malaria outbreak in Beitbridge District had resulted in one death in Beitbridge town during week 42 of 2017 [11]. Situated in Ward 6 of Beitbridge District, the Mtetengwe Clinic started to record a rapid increase in malaria cases during week 36, 2017 and subsequent weeks. Malaria threshold and alert levels for the clinic could not be calculated because the health facility was established in 2016, however malaria threshold levels for Makakabule Clinic which is in Ward 7 were used. Makakabule Clinic used to serve most of the population in Ward 6 before Mtetengwe Clinic was constructed.

This study aimed to investigate the malaria outbreak and determine the factors associated with contracting malaria in Ward 6 of Beitbridge. Understanding the risk factors associated with the occurrence of a malaria outbreak enables early and effective initiation of prevention interventions and control measures towards elimination of the disease in the affected area. The study also sought to investigate the malaria outbreak preparedness and response in the district. District health teams are responsible for detecting and responding to outbreaks at a local level. The assessment of the measures taken by the district health team to respond to a rise in malaria cases disease was imperative since the district was experiencing a second malaria outbreak; thus, this assessment could identify gaps in the malaria response system processes and make recommendations directed towards efficient and effective local disease outbreak response.

## Methods

An unmatched 1:1 case–control study was conducted in October 2017. This study employed the stages used in the investigation of an outbreak guidelines recommended by the Centre for Disease Control and Prevention (CDC) [12]. This is a guide to real time stepwise investigation of acute public health events at a local, national, or multinational level. The systematic approach was used in preparing for field work after establishing the existence of an outbreak, verifying a diagnosis to finding and recording cases before epidemiologically evaluating the study hypotheses. The results from the investigation were communicated to the authorities were they were used to implement control and preventive measures.

### Study setting

Beitbridge District is located 583 km south of the Zimbabwean capital, Harare, bordering Zimbabwe and South Africa. Mtetengwe Clinic is a rural health centre under the administration of Beitbridge Rural District Council, Matabeleland South Province, Zimbabwe. The clinic was opened in 2016 and it provides health services to Ward 6 population estimated at 3548. Mtetengwe Clinic covers villages like Mtetengwe, Mzingwane, Mapani, Tshinabazwimi, Malala and Bishopstone, BK Cawood, Beitbridge Juicing farming estates among others. Villagers and farm workers in this area live in house made of mud and wood or bricks roofed using thatch, asbestos or corrugated sheets.

The Ward 6 of Beitbridge District is primarily rural and it is located in an area of semi-arid land with erratic rainfalls and very hot climate. Due to poor annual rainfalls, the population depends on buying and selling of commodities purchased from South Africa. Furthermore, the district is also known for its thriving goat, cattle and game ranching activities. Beitbridge District offices are in Beitbridge town 22 km east of Ward 6. According to the Matabeleland South Generic Report of June 2017, the district had an estimated population of 128,454.

Entomological surveys carried out in Beitbridge District identified the main malaria vector in the area as *Anopheles gambiae* sensu lato (*s.l.)* and *Anopheles arabiensis.* However, the surveys also noted *Anopheles quadriannulatus* as a potential vector. This prompted the district to schedule indoor residual spraying (IRS) between the month of October and December every year depending on the availability of resources.

### Study population

The study population consisted of all adults and children residing in Ward 6 of Beitbridge District between week 36 and week 44 of 2017. The healthcare workers in the district were also included as key informants in the study. A case was a patient who resided within Ward 6, of Beitbridge District who presented with signs and symptoms of malaria and tested positive on rapid diagnostic test (RDT) for malaria from week 36 to week 44 of 2017. Symptoms of malaria referred to one or a combination of fever, vomiting, headache, general body malaise and rigors/chills. Controls who served as the comparison group were individuals who resided with or near a case and did not contract malaria during the period under study.

### Sampling

Using Stat-Cal embedded in Epi Info version 7.2.1.0 (CDC, USA) and assuming that living within a 3 km radius of a river or swamp was a significant risk factor for contracting malaria with an odds ratio (OR) of 2.7 and 43% of controls having been exposed [13], using a power of 80% and a 95% confidence interval (CI) gave the minimum required sample size of 66 cases and 66 controls. With expected 20% attrition rate the sample size was approximately equal to 83 cases and 83 controls.

The Mtetengwe Clinic line list which was used as the sampling frame for this study had 109 malaria cases and Ward 6 were purposively selected as study site in this investigation. Simple random sampling of cases was done by allocating numbers to all the 109 cases on the Mtetengwe Clinic line list and putting the numbered cards in a hat. After mixing, cards with numbers were blindly picked by a nurse from the clinic without replacement until the calculated sample size was reached. Controls were purposively selected from individuals residing with or within the neighbourhood of cases. To ensure that the control sample was representative enough, they were age-matched with cases.

For the assessment of the health workers used as key informants in the malaria outbreak response and preparedness in the district, maximum variation sampling was used. Different health workers with different roles in malaria epidemic investigation were interviewed at the district, facility and community level were utilized and this introduced a sample high heterogeneity while capturing maximum diversity of experiences [14]. The sample of key informants included purposively recruited district medical officer, district nursing officer, laboratory scientist, environmental health technician, and district pharmacist at the district level. The two nurses were interviewed at the facility level and three VHW were interviewed at the community level. All the key informants consented to participate for a 30 to 40 min interview.

### Data collection and analysis

Data collection was conducted over a period of 2 weeks using structured and pretested interviewer-administered questionnaires (Additional file 1). These were translated from English to the local language Shona and back translated to English to ensure comprehension of the questions. Treatment records, clinic registers as well as healthcare workers were part of the study population. The participant questionnaire aimed to determine the demographic characteristics, the knowledge levels on transmission of malaria and practices used for protection against the disease. Checklists were also used to assess for environmental health risk factors and the availability of resources essential to mount an outbreak response. Data collection tools were pretested using ten participants from a village in Gwanda District. The researchers carried out the malaria epidemic investigation from the beginning of week 44 to the end of week 46 of 2017. Local VHWs were used to assist in locating selected cases using the clinic line list during community visits which were conducted during week days from 8am to 4 pm. Appointments for interviewing key informants were made in advance to minimize inconveniences.

Data from hand-written copies were transferred into Microsoft Excel (Additional file 2) before it was exported into Epi Info version 7.2.1.0 (CDC, USA) for data cleaning, coding and analysis. The statistical software was used to calculate frequencies, means, proportions, odds ratios and p-values at 95% CI. Backward stepwise multivariate logistic regression analysis was done to control for any confounding variables.

Permission to conduct the study was sought from Provincial Medical Director of the Matabeleland South Province, and District Medical Officer Beitbridge District. Ethical review and approval were granted by the Africa University Research Ethics Committee and the approval number was 266/17. Written informed consent was obtained from the study participants before data was collected. Participation in this study was voluntary.

### Operational definitions

*Well-constructed house* a house made from bricks walls and a roof made from asbestos or corrugated roof [15].

Poorly constructed house a house made using mud and pole while the roof was either thatched with grass or non-thatched, that is made of asbestos or corrugated sheets. A house made of bricks and thatched with grass was classified under poorly constructed houses.

*Eaves* gaps between the top of the wall and the overhanging roof [15].

*Evening outdoor activities* habitually spending significant evening time outside the house from sunset to about 10 pm before going to bed.

## Results

### Description of the malaria outbreak by person

A total of 109 cases of malaria were reported from week 36 to week 44 of 2017 and no deaths occurred in the study area during this period. Four of the sampled cases could not be located since they had travelled outside Ward 6 and four refused to be interviewed thus, a total of 75 cases and 75 controls were interviewed of which 52% (n = 39) of the cases were females and the mean age of the malaria cases was 29 ± 13 years while most (35%) cases were in the age-group 20–9 years.

Study participants who had long lasting insecticide-treated nets (LLINs) (n = 85) reported that the LLINs were donated in 2015 and researchers noted that 45% of these had since developed holes which were potential entry points for the malaria vector. Of those who had LLINs, 26% (n = 22) did not use them because the treated nets were perceived to cause suffocation (n = 11), itchiness (n = 4), or individuals were not interested in using the nets (n = 7).

Although the community knowledge of malaria transmission was not significantly associated with contracting malaria, the majority (97%) managed to recall the use of LLINs while 35% mentioned IRS as a way of protecting self from malaria. 83% of participants agreed that one can protect self from getting malaria and all the participants reported that malaria disease is curable and they would visit the clinic upon suspecting any signs and symptoms of malaria. 93% of participants correctly identified that malaria is more common during the hot and wet season. 12% of the cases had travelled outside Ward 6 approximately 4 weeks before they were diagnosed of malaria while none of the controls had travelled outside Ward 6. The mean number of days taken from onset of malaria symptoms to visiting the clinic by the malaria cases was 3. Table 1 shows the sociodemographic characteristics of the study participants.

**Table 1:** Study participants’ socio-demographic information

Variable	Category	Cases n = 75 (%)	Controls n = 75 (%)
Gender	Male	36 (48)	33 (44)
	Female	39 (52)	42 (56)
Education level	None	9 (12)	10 (13)
	Primary	43 (57)	42 (56)
	Secondary	23 (31)	23 (31)
Income source	Dependent	21 (28)	22 (29)
	Formal	15 (20)	15 (20)
	Informal/self	39 (52)	38 (51)
Religion	Apostolic	12 (16)	21 (28)
	Pentecostal	12 (16)	13 (17)
	Protestant	15 (20)	8 (11)
	None	36 (48)	33 (44)

### Description of the malaria outbreak by place

The residents of Ward 6 of Beitbridge District mainly lived in rural villages and farm compounds where the walls of the houses were made from bricks or mud and pole while roofing material was mainly thatch, asbestos or corrugated zinc sheets. 36%, 32%, 29% and 3% of the participants resided in houses made from brick and asbestos/corrugated sheets, mud and thatch, brick and thatch, and mud and asbestos/corrugated sheets, respectively.

**Fig. 1:**
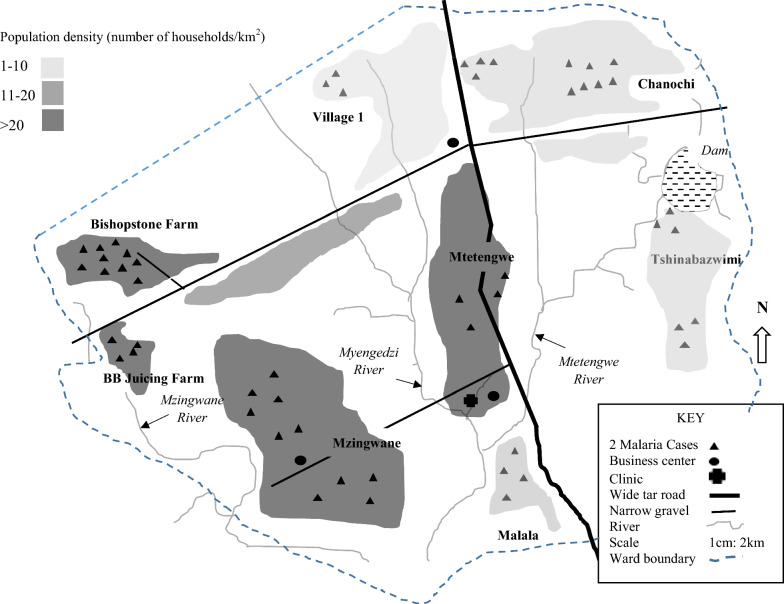
The spot map of Ward 6 of Beitbridge District showing the distribution of the 109 cases for week 36 to week 44 of 2017 according to the Mtetengwe Clinic line list

Although 45% of the participants slept in houses with conventional windows, some of the windows did not have panes or the panes were broken exposing the inhabitants to mosquitoes. The majority of malaria cases from the clinic line list for week 36 to week 44 were from Bishopstone farm compound (18%; n = 20) and Mzingwane village (17%; n = 19). Figure 1 shows the spot map of Ward 6 of Beitbridge District showing the distribution of the 109 cases for week 36 to week 44 of 2017 according to the Mtetengwe Clinic line list. 33% (n = 49) of the participants had IRS done more than 8 months earlier and 47% (n = 23) of these were malaria cases.

### Description of the malaria outbreak by time

**Fig. 2:**
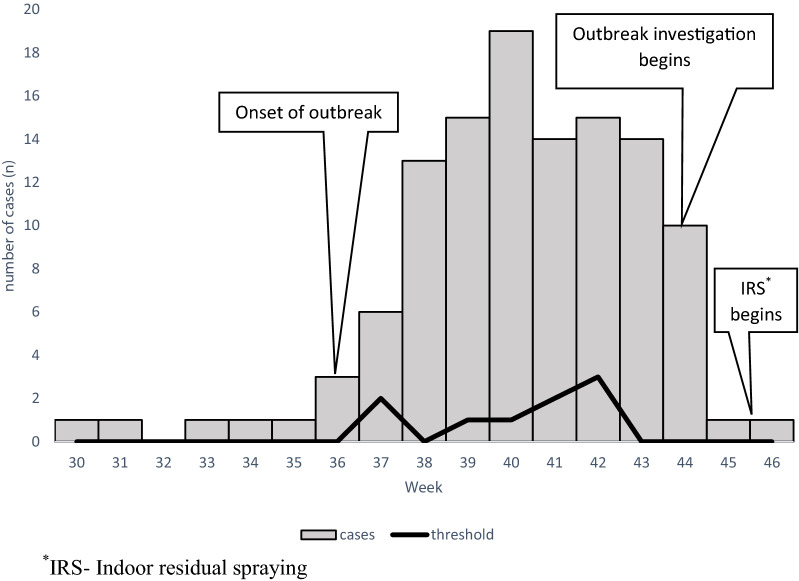
An epidemiological curve for malaria outbreak in Ward 6 of Beitbridge District, week 36 to week 44, 2017

Figure 2 displays an epidemiological curve reporting the gradual increase in number of individuals who presented with clinical symptoms of malaria and tested positive for the disease. The curve shows a common source outbreak with intermittent exposure. The irregular peaks represents the timing and extent of exposure to the malaria parasite. Malaria positive cases started to increase gradually from the 9th of September 2017, peaked on the 3rd of October 2017 and steadily began to decline thereafter. Although malaria cases identification continued after the outbreak period, the frequencies ranged below the threshold level.

### Factors associated with malaria transmission

Table 2 shows the bivariate analysis of factors associated with contracting malaria in Ward 6, Beitbridge District between week 36 and week 44 of 2017.

**Table 2:** Bivariate analysis for factors associated with contracting malaria in Ward 6 of Beitbridge District for week 36 to Week 44, 2017

Variable	Category	Cases	Controls	OR	95% CI	p-value
Gender	Male	36	33	0.85	0.45–1.62	0.62
	Female	39	42			
Education	None/primary	52	52	1	0.50–2.0	1
	Secondary	23	23			
Age (years)	≥ 20	54	61	0.59	0.27–1.27	0.18
	< 20	21	14			
Income status	Employed	54	53	1.07	0.53–2.17	0.86
	Dependent	21	22			
Religion	Apostolic	13	21	0.54	0.25–1.18	0.12
	Non-apostolic	63	54			
Village/farm	Mzi/Bishopstone	35	38	0.85	0.45–1.62	0.62
	Other	40	37			
House had visible open eaves	Yes	60	43	2.97	1.44–6.16	0.0028*
	No	15	32			
Residents closed eaves before sunset	No	15	20	0.45	0.20–1.02	0.055*
	Yes	45	27			
House has conventional windows	No	40	34	1.38	0.73–2.62	0.60
	Yes	35	41			
Sleeping in a poorly constructed house	Yes	64	43	4.33	1.97–9.51	0.000*
	No^a^	11	32			
Has LLINs	No	27	38	0.55	0.28–1.05	0.07
	Yes	48	37			
Slept under LLIN last night	No	10	9	0.81	0.29–2.28	0.70
	Yes	38	28			
Wearing long clothes at night	No	67	60	2.10	0.83–5.29	0.11
	Yes	8	15			
Spent evenings	Outdoors	62	51	2.24	1.04–4.85	0.037*
	Indoors	13	24			
Lived < 1 km from water source	Yes	44	36	1.54	0.81–2.93	0.19
	No	31	39			
IRS done in last 8 months	No	52	49	1.20	0.61–2.38	0.60
	Yes	23	26			
History of traveling outside Ward 6	Yes	12	0	0		
	No	63	75			

LLTNs: Long-lasting insecticide-treated nets; IRS: Indoor residual spraying; Mzi/Bishopstone: Mzingwane village or Bishopstone Farm

^a^Sleeping in a house made from bricks walls and a roof made from asbestos or corrugated roof

*Statistically significant p-value

### Multivariate analysis

Backward stepwise regression analysis was conducted to estimate the variables associated with contracting malaria while controlling for confounding factors. Only variables with p < 0.1 in bivariate analysis were used in constructing a mathematical model to describe the association between exposure and disease and other variables that may confound the effect of the exposure. The results of the logistic regression are presented in Table 3. While controlling for the presence of open eaves and having an LLIN, sleeping in a poorly constructed house remained statistically significant. Thus, individuals who slept in a poorly constructed houses were three times more likely to contract malaria than individuals sleeping in a well-constructed house. To control for multicollinearity while reducing omitted variable bias, the variable ‘house had visible eaves was excluded from the regression analysis because it had high partial correlations which inflated standard errors for ‘closing eaves at sunset’ and ‘sleeping in a poorly constructed house’.

**Table 3:** Multivariate analysis of the factors associated with contracting malaria in Ward 6, Beitbridge District for week 36 to week 44, 2017

Variable	Coefficient	AOR	95% CI	p-value
Spending the evening outdoors	0.80	2.23	0.81–6.10	0.12
Closing eaves at sunset	− 0.64	0.53	0.21–1.28	0.16
Having an LLIN	− 0.36	0.70	0.30–1.61	0.40
Sleeping in a poorly constructed house	2.13	8.40	1.69–41.66	0.009*

*p > 0.05, result is statistically significant

### Malaria outbreak preparedness and response

Before the outbreak, malaria cases were being reported to the district office on a weekly basis. The clinic staff notified the District Health Team (DHT) of the sudden increase in malaria cases on the 9th of September, 2017. The Emergency Preparedness Response (EPR) team which used to converge every Wednesday, met immediately and recommended mobilization of resources in preparation for a potential malaria outbreak. An Environmental Health Technician (EHT) from Makakabule Clinic and 2 Laboratory technicians from the district office were deployed by the DHT to establish the possibility of an outbreak in Ward 6 within 48 h since there was no EHT at Mtetengwe Clinic. The clinic staff collected blood specimens from cases identified by RDT and the blood specimens were collected daily from the clinic to the district laboratory.

Mtetengwe Clinic had a staff complement of two primary care nurses and one community-hired nurse aide. All the nurses at the clinic were trained in malaria surveillance and response as well as malaria case management. Utilizing the epidemic preparedness and rapid response guidelines, the nurses at Mtetengwe Rural Health Centre monitored malaria trends and drafted malaria threshold graphs. Mtetengwe Clinic did not experience stock outs of supplies and anti-malarial medications during the period of the outbreak.

The EHT and local VHWs, conducted active case finding by visiting the surrounding communities, identifying risk factors and giving information on malaria. Health education and promotion on use of LLINs and early seeking of treatment was ongoing. Some malaria positive cases were accompanied by the Village Health Worker to the clinic. Farm owners from distant farms such as BK Cawood and Bishopstone offered free transport for ill workers and their relatives. Indoor residual spraying was conducted in the area during the last 2 weeks of November 2017. The district team was in constant contact with the clinic staff throughout the outbreak period and the outbreak was declared over on the 20th of November 2017.

### Malaria case management

Due to the unavailability of microscopy tests and microscopists in the rural areas, health workers relied on malaria rapid diagnostic test (RDT) to confirm diagnosis and this test can be conducted by nurses and village health workers (VHWs). The VHWs were equipped with RDT kits for rapid testing as well as artemisinin-based combination therapy (ACT) for uncomplicated cases. Uncomplicated cases who visited the clinic were also given ACT with the first dose being directly observed by a healthcare worker before they were discharged home. All cases reported to have completed the full course of anti-malarial medication. The complicated cases were referred to Beitbridge District Hospital for further management. The two cases who were referred during this study period, recovered well. At Mtetengwe Clinic, malaria treatment and diagnostic testing is free for all age groups.

## Discussion

This study sought to investigate a malaria outbreak, determine the factors associated with contracting malaria and to investigate the malaria epidemic preparedness and response in Ward 6 of Beitbridge District. The majority of the malaria cases were between the ages of 20–30 years which is contrary to the findings of the study by Drakeley et al. in which the disease was more prevalent in the age group 5–19 years [16]. This may be attributed to the young population in the study area as evidenced by the proportion of cases decreasing with age. Many people come to Ward 6 for economic opportunities particularly seeking employment in the farming estates and selling goods from South Africa. Most of the farmworkers and traders are between the ages of 20 to 30 years.

This study revealed that sleeping in a poorly constructed house were significantly associated with contracting malaria while closing eaves was protective. A similar study in Gambia reported that closing eaves halved the prevalence of malaria caused malaria in children [17]. A systematic study of five case–control studies and two cohort studies showed that both living in houses made of brick walls and closing eaves reduced the odds of contracting malaria infection by a quarter [18]. Well-constructed houses protect inhabitants from malaria by preventing entry of the malaria vectors. Modern brick walls and roofs also limit resting places for mosquitoes and reduce the attractiveness of the internal environment to the vectors when compared to the traditional mud/pole and thatch houses [18]. Although the use of LLINs and IRS are equally important, housing improvements have significantly contributed to a decline in malaria cases and elimination of the disease in many countries [19, 20]. Two studies on malaria outbreak in Zimbabwe also cited that the presence of open eaves increased the likelihood of contracting malaria [13, 21]. The majority of the remote Ward 6 residents lived in poorly constructed houses and similar settings of poverty and remoteness were found to be risky with regards to contracting malaria in Amazon Villages [22].

Spending time outdoors after sunset was significantly associated with contracting malaria and this finding was similar to another study in which overnight hunting and spending night time outdoors were significant malaria transmission risk factors in French Guiana [23]. Zimbabwean studies in Chipinge and Mberengwa also cited that routine outdoor activities such as bathing and night fishing were significantly associated with contracting malaria [13, 24]. Mothers and children in Ward 6 were known to spend evening time outdoors sitting around a fire, cooking and exposing themselves to mosquito bites thus increasing their chances of contracting malaria. Adult males particularly those who were employed at the farms were also exposed to mosquito bites since they dismissed well after sunset.

The community had fairly good knowledge of malaria disease since the majority of participants managed to identify infected mosquitoes as the vector for the disease although a few still believed that eating infected fruits cause malaria. Most of the cases and controls could identify the signs and symptoms of malaria and all cases reported to have completed the treatment course. A similar study done also recorded high knowledge of the signs and symptoms as well as prevention of the disease [13]. This finding was also consistent with the malaria survey which also cited reasonably good knowledge about malaria causes, signs and symptoms and prevention among the rural community of Zimbabwe [25].

Participants reported that malaria disease was curable and they would visit a healthcare centre upon suspecting that they had the disease. Health-seeking behaviour in Ward 6 was good, a finding that was contrary to what Onwjekwe and colleagues, found out in Nigeria were populations consulted herbalists, used spiritual/ritual water or just pray ignoring the malaria symptoms [26]. This difference in results can be attributed to increased sensitization on the disease in Beitbridge District since this was a second malaria outbreak within the same year.

Having an LLIN does not directly translates to its consistent use in malaria prevention; this was noted in 56% of LLIN owners being malaria cases. A study by Msellemu et al. in Tanzania also made the same conclusion after finding out that LLINs were often reserved for children and visitors exposing other family members to the vector [27]. The study participants who had LLINs in Ward 6 reported that the LLINs were donated in 2015 and on observation some nets had developed holes. Such damages to the preventive barrier can still expose individuals to mosquito even though they used the nets. Some LLIN owners mentioned that the nets were suffocating or caused itchiness as barriers to LLIN use. This resulted in inconsistencies in the use of nets as malaria preventive measures. The fact that some of the controls who had LLINs did not use them reveals the idea that mosquito-proof housing provides some form of protection that does not depend on human compliance.

The use of EPR team to mount a response against the outbreak was an effective way of combating the malaria outbreak. Unlike in 2015 when the Beitbridge Emergency Response team did not consistently conduct EPR meetings [21], the team was sitting every Wednesday for the past 6 months and there were meeting minutes to support this. All the nurses at Mtetengwe Clinic were trained in integrated disease surveillance and response, a finding that was contrary to a similar study done in Shamva, Zimbabwe [9]. Repeated malaria outbreak attacks had prepared the Beitbridge District team to be on high alert for a possible increase in cases of the disease. This encouraged prompt outbreak notification and response to prevent further proliferation of the disease in Ward 6. Overall, both the healthcare centre and district were well prepared to respond to this malaria outbreak because most essential stocks were available and there were no stock outs during the course of the outbreak.

Zimbabwe faces a critical shortage of human resource for health and the shortage of nurses, EHTs and microscopists can have a negative impact on the control of malaria in Zimbabwe [9]. The EHT deployed during the current outbreak was from another clinic while Mtetengwe Clinic had a vacant EHT post due to the recruitment freeze on health posts [28]. The cadre was evidently overwhelmed with responsibility and rarely had time to cover Mtetengwe Clinic. This may have resulted in the insidious increase of cases in the study area.

### Study limitations

All cases used in this study were diagnosed using RDT and this may have resulted in recruitment of participants infected outside the duration of the outbreak particularly during the early stages of the outbreak. Self-reported data used in this study might have introduced recall bias on exposure as well as social desirability when responding to interview questions. The sample size for the study was fairly small which could affect the power to identify significant changes in some exposure variables. This study did not include those patients who had a positive malaria test, received ACT from local VHW and recovered before visiting the clinic. The study also focused on a single infection assessment done once per person without repeat screening which limits the inference of findings to the actual malaria infections.

## Conclusion

Health promotion messages should emphasize the importance of closing eaves and other entry points of the malaria vector, and constructing better houses in the future. Resident should also be educated on the importance of consistent use of LLINs. The district had to improve on preventive measures like distribution of LLINs and lobby for more human resources to assist with malaria surveillance thus, curbing the recurrence of malaria outbreaks in the future. To counter the possibility of another malaria outbreak, these measures had to be instituted promptly.

## Supplementary information

**Additional file 1.** Participant questionnaire used for data collection in the study.

**Additional file 2.** Dataset used for data analysis in the study.

## Data Availability

The datasets supporting the conclusions of this article are included within the article (and its additional files)

## Abbreviations

ACT: Artemisinin-based combination therapy
AOR: Adjusted odds ratios
CI: Confidence Interval
DHT: District Health Team
EHT: Environmental Health Technician
EPR: Emergency preparedness and response
LLINs: Long-lasting insecticide-treated nets
OR: Odds ratios
RDT: Rapid diagnostic test
VHWs: Village Health Workers
WHO: World Health Organization
