# Supplementary material for: Malaria outbreak investigation in a rural area south of Zimbabwe: a case–control study
Source: Malar J. 2020 Jun 1;19:197. doi: 10.1186/s12936-020-03270-0 (PMC7268448; doi:10.1186/s12936-020-03270-0)
Supplement: Supplementary file 1 — Additional file 1. Participant questionnaire used for data collection in the study. [file 12936_2020_3270_MOESM1_ESM.docx]

**Questionnaire for the Malaria Outbreak Investigation in Ward 6, Beitbridge District**

My name Paddington Mundagowa and I am carrying out a study to identify the factors associated with contracting malaria in Ward 6, Beitbridge District. The purpose of the research is to identify factors that put you at risk of getting malaria. This will assist in coming up with effective prevention and control strategies to prevent future outbreaks. Your participation in this study is voluntary and the information collected from you is confidential. If you agree to participate please tick 1 and if you do not agree please tick 2.

[ ] 1 [ ] 2 Date: _____/________/__________

Questionnaire Number: [ ]

**Section A: Demographic data**

[ ] Case [ ] Control

1. How old are you? (Completed years)__________________

2. Gender: [ ] Male [ ] Female

3. Place of residence: ______________________________Village/ Farm.

4. What is the highest level of education did you attain?

[ ] Never went to school [ ] Primary [ ] Secondary [ ] Tertiary

5. What is your Religion? [ ] Protestant [ ] Traditional [ ] Pentecostal [ ] Muslim [ ] None

6. What is your major source of income?

[ ] Formal employment [ ] Informal employment/self-employed [ ] Dependent

**Section B: Knowledge about malaria for cases and controls**

7. How is malaria transmitted?

[ ] Eating infected fruits [ ] Evil spirits [ ] Infected mosquito bites [ ] sexual contact

8. What season are you most likely to have malaria?

[ ] Hot & wet season [ ] Cold & dry season

9. What are the signs and symptoms of malaria?

[ ] Fever [ ] headache [ ] shivering [ ] Sweating

[ ] General body weakness [ ] Muscle and joint pains

[ ] nausea and vomiting

10. Can you protect self from getting malaria? [ ] Yes [ ] No

11. If 10 is YES, How do one protect self from getting malaria?

(*Let participant mention the ways without giving them the answers for Yes or No*)

[ ] sleeping under a mosquito net [ ] using a mosquito repellent

[ ] indoor residual spraying of mosquitoes [ ] burning a mosquito coil

[ ] closing widows early at sunset [ ] burning dung [ ] fill stagnant water sources

12. Where do mosquitoes usually breed? [ ] Stagnant water [ ] in running water

13. Is malaria curable? [ ] Yes [ ] No

14. What do you do when you suspect that you have malaria?

[ ] Visit the clinic [ ] go to a traditional healer

[ ] Visit a faith healer [ ] use traditional medicine

15. When taking malaria treatment course, when should you stop treatment?

[ ] after completing the course [ ] You can stop when symptoms disappear

**Section C: Factors associated with contracting malaria for cases and controls**

16. What kind of a house do you sleep in? (*Observe and confirm*)

[ ] Brick & thatch [ ] brick & asbestos/iron sheets

[ ] pole, mud & thatch [ ] pole & mud & iron sheets/asbestos

16b. Type of house (*By observation*) [ ] poorly built [ ] well built

17. Does your house that you sleep in have open eaves? (*Observe and confirm)*

[ ] Yes [ ] No

18. If yes, do you close the eaves at night? [ ] Yes [ ] No

19. Does your house that you sleep in have conventional windows? (*Observe*) [ ] Yes [ ] No

20. If 19 is yes, what time do you close the windows?

[ ] Just before sunset [ ] Never closed

[ ] Never opened [ ] sometimes we don’t close the windows

21. Was your house sprayed (IRS) in the past 8 months? [ ] Yes [ ] No

22. Do you leave near (1km radius) a stagnant water body (dam, swamp)? (*Observe)*

[ ] Yes [ ] No

23. Do you wear clothes with long sleeves and long trousers at night? [ ] Yes [ ] No

24. Where do you usually spent evening times? [ ] Outdoors [ ] Indoors

25. Do you have a mosquito net/s? (*Ask to check,* *Observe and confirm*) [ ] Yes [ ] No

26. If YES, does everyone sleep under a mosquito net (is number of sleeping spaces equal to number of mosquito nets)? [ ] Yes [ ] No

27. Did you sleep under a mosquito net last night? [ ] Yes [ ] No

28. How often do you sleep under the mosquito net? [ ] Always [ ] sometimes [ ] never

29. If never or sometimes, why?

[ ] it’s itchy [ ] it’s hot and suffocating

[ ] it causes cancer [ ] I am just not interested

30. What other alternatives measures do you use to prevent yourself from being bitten by mosquitoes? [ ] mosquito coil [ ] conventional repellents [ ] burn dung [ ] nothing

31. Did you travel outside Ward 6 during the four weeks before you got ill? [ ] Yes [ ] No

**Section D: Case Management-FOR CASES ONLY**

32. How many days did it take you to visit the clinic? Number of Days: ___________________

33. Which of these signs and symptoms of malaria did you experience?

[ ] fever [ ] shivering [ ] sweating [ ] headache

[ ] general body weakness [ ] muscle and joint pains [ ] nausea and vomiting

34. Before you visited the hospital/clinic, what did you take to ease the symptoms?

[ ] None [ ] Herbal medicine [ ] Pain Killer [ ] Anointing oil/holy water

[ ] other (specify) ___________________________________

35. Did you complete your malaria treatment course? [ ] Yes [ ] No

36. For how long were you ill after starting the malaria course?

[ ] 1-3 days [ ] 4 days-1week [ ] >1 week

**THANK YOU FOR PARTICIPATING IN THE STUDY**
