# Supplementary material for: Malaria outbreak investigation in a rural area south of Zimbabwe: a case–control study
Source: Malar J. 2020 Jun 1;19:197. doi: 10.1186/s12936-020-03270-0 (PMC7268448; doi:10.1186/s12936-020-03270-0)
Supplement: Supplementary file 2 — Additional file 2. Dataset used for data analysis in the study. [file 12936_2020_3270_MOESM2_ESM.docx]

This study used Stat-Cal that is embedded in Epi Info version 7.2.1.0 (CDC, USA). We assumed that living within a 3 kilometre radius of a river or swamp was a significant risk factor for contracting malaria with an odds ratio of 2.7 and 43% of controls having been exposed using findings from Kureya et al, (2017) and a power of 80% and a 95% confidence interval gave the minimum required sample size of 66 cases and 66 controls (Fleiss).

With expected 20% attrition rate: 66/0.8 =82.5 which is approximately 83.

Therefore the maximum calculated sample size was equal to 83 cases and 83 controls.
